# Deficiency of N1-Adenine Methyltransferase Aggravates RNA and Protein Aggregation

**DOI:** 10.3390/cells14171370

**Published:** 2025-09-02

**Authors:** Marion Alriquet, Roberto Arsiè, Giulia Calloni, Gian G. Tartaglia, R. Martin Vabulas

**Affiliations:** 1 Buchmann Institute for Molecular Life Sciences, Goethe University Frankfurt, 60438 Frankfurt am Main, Germany; 2 Institute of Biochemistry, Charité-Universitätsmedizin Berlin, 10117 Berlin, Germany; roberto.arsie@charite.de; 3 Centre for Human Technologies (CHT), RNA System Biology Lab, Istituto Italiano di Tecnologia, 16152 Genoa, Italy; gian.tartaglia@iit.it

**Keywords:** TRMT6/TRMT61A, N1-methyladenine, RBP, protein aggregation

## Abstract

RNA modifications regulate diverse aspects of transcripts’ function and stability. Among these, N1-methyladenine (m^1^A) is a reversible mark primarily installed by the TRMT6/TRMT61A methyltransferase on tRNA, though it is also found on other RNA types. m^1^A has been implicated in protecting mRNAs during acute protein misfolding stress. However, the role of m^1^A under chronic proteotoxic conditions, such as intracellular amyloid aggregation, remains poorly understood. To address this gap, we examined the effects of reduced N1-adenine methylation in human cells undergoing amyloidogenesis. Suppression of the methyltransferase TRMT61A or overexpression of the m^1^A-specific demethylase ALKBH3 enhanced amyloid aggregation. A deficiency of N1-adenine methylation also impaired the expression of a reporter mRNA-encoded protein, highlighting the protective role of m^1^A in safeguarding transcript functionality. Proteomic analysis of amyloid aggregates from TRMT61A-deficient cells revealed increased co-aggregation of bystander proteins, particularly those with known RNA-binding activity. At the same time, the aggregates from methylation-deficient cells contained elevated levels of mRNAs. Collectively, our findings support a role for m^1^A in preventing RNA entanglement within aggregates and limiting RNA-mediated propagation of protein co-aggregation.

## 1. Introduction

Eukaryotic cells rely on a complex network of specialized proteins to maintain proteome homeostasis (proteostasis) under various conditions [1]. This network enables cells to remain functional during both acute and chronic stress. Disruption of proteostasis can lead to the accumulation of misfolded and aggregated proteins, contributing to the pathogenesis of neurodegenerative diseases such as Alzheimer’s or Parkinson’s. Recently, increasing attention has been directed toward understanding how other cellular components—such as lipids and nucleic acids—contribute to proteome stability.

To date, more than 170 distinct RNA modifications have been identified in living organisms [2]. These modifications influence the post-transcriptional fate of transcripts through diverse mechanisms, including the modulation of RNA stability, regulation of RNA-binding protein (RBP) interactions, and alteration of inter- and intramolecular associations [3]. N1-methyladenine (m^1^A), one such modification, was first discovered in tRNA [4], where it is installed by the TRMT6/TRMT61A (tRNA (adenine(58)-N(1))-methyltransferase) complex [5]. Within this complex, TRMT61A serves as the catalytically active “writer” subunit, utilizing S-adenosyl methionine (SAM) as the methyl donor. Although the TRMT6 subunit lacks SAM-binding site, it contributes to substrate recognition and specificity through extensive interactions with the tRNA molecule [6]. In addition to TRMT6/61A, other, less-characterized m^1^A writers include the mitochondrial TRMT61B, several Trm10 family methyltransferases, and nucleomethylin.

The m^1^A modification is reversible and methyl can be removed by “eraser” demethylases from the ALKBH (alkylation B homolog) family of α-ketoglutarate-dependent dioxygenases. These enzymes act on both DNA and RNA to remove functional or deleterious alkyl groups, thereby preserving genome and transcriptome integrity [7]. In humans, ALKBH1, ALKBH3, ALKBH7, and FTO (fat mass and obesity-associated protein) have been implicated in demethylating N1-methyladenine [6].

While m^1^A has been long associated with tRNA stability and translation control, emerging evidence suggests that it also plays important role in mRNA [8,9]. During acute cellular stress, m^1^A levels are markedly elevated in stress granules (SGs), and m^1^A motif-containing reporter transcripts are functionally protected under those conditions [10]. These findings raise the possibility that m^1^A may have a protective role also during chronic proteotoxic stress, such as amyloidogenesis. Protein aggregation is frequently accompanied by changes in RNA metabolism, yet the functional contribution of specific RNA modifications—whether causal or protective—remains unclear [11,12].

In this study, we explored the role of the TRMT6/TRMT61A complex and m^1^A modification during amyloidogenesis induced by the transient overexpression of the amyloid-β 1–42 peptide, a key pathogenic component in Alzheimer’s disease. Using m^1^A-sensitive mRNA reporter constructs, mass spectrometry, and microscopy-based protein aggregation assays, we demonstrate that the N1-adenine modification machinery plays a protective role in mitigating amyloid formation and the associated co-aggregation of cellular proteins. Our findings support a model in which insufficient m^1^A tagging exacerbates proteome damage during chronic proteostasis stress.

## 2. Materials and Methods

### 2.1. Reagents, Plasmids, Antibodies

TRIzol was purchased from Invitrogen (Carlsbad, CA, USA). All other chemicals were from Sigma-Aldrich (St. Louis, MO, USA) unless otherwise specified. Aβ-GFP, Flag-tagged TRMT6, and TRMT61A mammalian expression vectors were purchased from Genscript (Piscataway, NJ, USA). The following antibodies were used: anti-GFP (1814460) from Roche (Basel, Switzerland); anti-TRMT6 (A303-008A-M) from Bethyl (Montgomery, TX, USA); anti-TRMT61A (sc-107105) from Santa Cruz Biotechnology (Dallas, TX, USA); HRP-conjugated anti-rabbit-IgG (7074) and Alexa Fluor 647-conjugated anti-rabbit IgG (4414) from Cell Signaling (Danvers, MA, USA); anti-Flag (F1804) and HRP-conjugated anti-mouse IgG 151 (A9044) from Sigma-Aldrich.

### 2.2. Constructs

Plasmids generated for this study were prepared by standard molecular biology techniques, and all coding sequences were verified by sequencing. Three FLAG tags were inserted into the TRMT61A expression vector. A 3xFLAG-NQO1 eukaryotic expression vector was modified to introduce an m^1^A motif upstream of the coding sequence to generate the WT-NQO1 reporter. To construct the MUT-NQO1 reporter, the methylated adenine within this motif was mutated to uracil.

### 2.3. Cell Lines

The HeLa human adenocarcinoma cell line (ATCC #CCL-2) and the B16 murine melanoma cell line, subclone F0 (ATCC #CRL-6322) were obtained from the American Type Culture Collection (ATCC, Manassas, VA, USA). HeLa wild-type (WT) and TRMT61A knockdown (KD) cell lines were cultured in DMEM (Gibco, Waltham, MA, USA) supplemented with 10% (vol/vol) FBS (Gibco), 2 mM L-glutamine (Roth, Karlsruhe, Germany), 100 U/mL Penicillin, 100 µg/mL streptomycin, 1 g/L glucose, 1 mM sodium pyruvate, and 1× non-essential amino acids (NEAA, Gibco) at 37 °C in a humidified incubator with 5% CO_2_. TRMT61A KD HeLa cells were generated from HeLa WT cells by transfection with 1 μg TRM61A Double Nickase Plasmids (h) mix (Santa Cruz, sc-408677-NIC) followed by puromycin selection. Cell lines were routinely tested for *Mycoplasma* contamination using the Venor^®^GeM OneStep kit (Minerva Biolabs, Berlin, Germany).

The B16-F10 cell line was cultured in DMEM supplemented with 10% (vol/vol) FBS, 2 mM L-glutamine, 100 IU/mL penicillin G, 100 μg/mL streptomycin sulfate, and 1× nonessential amino acids, under the same incubation conditions.

### 2.4. Western Blotting

Samples were lysed in 1× Laemmli Sample Buffer (62.5 mM Tris base, 2% SDS, 10% glycerol, 5% 2-mercaptoethanol, 0.01% bromophenol blue) and sonicated on ice. After boiling for 5 min at 95 °C, lysates were loaded on a 12% polyacrylamide gel (15 min 80 V, followed by 1 h at 150 V). Separated proteins were then transferred onto methanol-activated PDVF membrane using a semi-dry apparatus (1 h at 22 V) or a wet transfer system (90 min at 100 V). Membranes were blocked in 5% skim milk in TBST for 1 h at room temperature, probed with primary antibodies overnight at 4 °C, washed three times in TBST (5 min each, room temperature), and then probed with the appropriate secondary antibody for 1 h at room temperature. After three additional TBST washes, membranes were developed using SuperSignal West Pico PLUS (Thermo Fisher Scientific, Waltham, MA, USA) or WesternBright chemiluminescence substrate Sirius (Biozym, Oldendorf, Germany). Chemiluminescence images were acquired using a ChemiDoc MP (Bio-Rad, Hercules, CA, USA) or Fusion FX (Vilber Lourmat, Marne-la-Vallée, France) imaging system, and band intensities were quantified using Image Lab 5.0 (Bio-Rad) or Fiji (ImageJ v1.54f).

### 2.5. Transfections and Immunofluorescence

For Aβ aggregation assays, 4 × 10^6^ HeLa cells were electroporated with 30 μg of either Aβ-GFP or GFP expression vectors and seeded onto poly-L-lysine-coated coverslips in a 12-well plates at 5 × 10^5^ cells/well. The culture medium was refreshed after 6 h. The cells were processed for microscopy or collected and lysed with SDS sample buffer for Western blotting 48 h after transfection. For immunofluorescence, cells were fixed with 4% PFA for 1 h at room temperature and stained with DAPI. Cells were imaged using a Zeiss LSM-780 inverted confocal microscope (Oberkochen, Germany) with a 40× oil immersion objective. Using CellProfiler 3.0 (Cambridge, MA, USA), nuclei were detected and cells identified by propagation from the nuclei. Fluorescence intensity was measured for each cell. Dead or dying cells were excluded based on intensity thresholds. The experiment was performed in triplicate and at least 200 GFP-positive cells were analyzed per condition and per repetition to determine the fraction of cells containing aggregates. For methylation reconstitution, 8 × 10^6^ HeLa TRMT61A KD cells were transfected by electroporation with 10 μg Aβ-GFP with or without 20 μg FLAG-TRMT6 and 20 μg 3xFFLAG-TRMT61A. The cells were lysed 48 h after transfection.

For analysis of the enzymatically impaired TRMT61A mutant D181A, HeLa TRMT61A KD cells were seeded in 12-well plates at a density of 1 × 10^5^ cells/well. The next day, cells were co-transfected with 1 μg Aβ-GFP and either 1 or 2 μg TRMT61A (WT or D181A) using polyethylenimine (DNA–PEI ratio was 1:6 using 1 mg/mL PEI solution). Cells were lysed 24 h after transfection. For Aβ aggregation upon NQO1 reporter transfection, approximately 4 × 10^6^ HeLa WT cells were electroporated (300 V, 950 µF) with 15 µg Aβ-GFP plasmid together with 15 µg either a WT-NQO1 or MUT-NQO1 reporter (control sample: empty vector) in 400µL intracellular buffer (120 mM KCl, 0.15 mM CaCl_2_, 10 mM K_2_HPO_4_/KH_2_PO_4_, pH 7.6, 25 mM Hepes, 2 mM EGTA, 5 mM MgCl_2_) [13]. After 2 min on ice, cells were plated in a 6-well plates containing poly-L-lysine-coated coverslips. After 24 h, cells were washed with DPBS and fixed for 10 min at room temperature with freshly prepared 4% PFA. Samples were then washed twice in DPBS and mounted on a coverslip with ROTI^®^Mount FluorCare DAPI (Roth). Imaging was performed on a Nikon SoRa Spinning Disk Confocal CSU-W1 microscope (Melville, NY, USA). Counting of fluorescent cells and aggregates was carried out manually using the Fiji (ImageJ v1.54f). For ALKBH3 overexpression, HeLa WT cells were electroporated (300 V, 950 µF) with 5 µg Aβ-GFP and 20 µg ALKBH3 plasmids. Cells were incubated for 48 h on poly-L-lysine-coated coverslips (for immunofluorescence) or in 6-well plates (for protein quantification). After washing with DPBS, cells were fixed with 4% PFA at room temperature and mounted on a cover slip with DAPI. Sample were visualized using a Nikon SoRa Spinning Disk Confocal CSU-W1 microscope. For protein quantification, cells were lysed in 1× Laemmli sample buffer, sonicated, and analyzed by Western blot.

### 2.6. Quantitative PCR

Total RNA was extracted using TRIzol reagent. RNA was reverse transcribed with RevertAid kit (Thermo Fisher Scientific) and diluted 10-fold. NQO1 reporters and GAPDH cDNAs were amplified using respective primers and KiCqStart SYBR Green qPCR ReadyMix (Merck, Darmstadt, Germany). Cycling conditions were 3 min at 95 °C, followed by 39 cycles of 15 s at 95 °C, 30 s at 58 °C, and 15 s at 72 °C. Each reaction was carried out in triplicate, and results were analyzed as previously described [14], with GAPDH used as an internal control. Data analysis was performed using CFX Manager Software 3.1 (Bio-Rad).

### 2.7. WT-NQO1 and MUT-NQO1 Reporter Assay

HeLa cells were seeded in 12-well plates at a density of 1 ×10^5^ cells per well. The next day, cells were transfected with 250 ng reporter constructs together with varying amounts of Aβ-GFP construct (0, 250, 500, or 1000 ng) using PEI (DNA–PEI ratio 1:6 using 1 mg/mL PEI solution). 24 h after transfection, cells were harvested and analyzed by SDS-PAGE and Western blotting using anti-GFP and anti-GAPDH antibodies. For quantitative comparison, the NQO1 amount per unit of Aβ-GFP was calculated by multiplying the respective NQO1 and Aβ-GFP signal values. Then, the average for all three Aβ-GFP concentrations was calculated. The average for the WT-NQO1 reporter was set to 1.

### 2.8. RNA FISH

The RNA FISH probe was prepared using the Red FISH Tag™ RNA Kit (Invitrogen) according to the manufacturer’s instructions. Briefly, a pcDNA3 vector containing the NQO1 gene was linearized and subjected to in vitro transcription with amine-modified UTPs. The resulting RNA was purified and conjugated to an amine-reactive dye (Alexa Fluor^®^ 594). After purification, the probe was diluted in FISH fixation buffer (2× SSC buffer, 10% formamide, 10% dextran sulfate, 0.5 U/µL RNase inhibitors) for subsequent use. Cells were electroporated and fixed on a poly-L-lysine-coated glass after 24 h, as described above. After fixation, samples were permeabilized with 0.2% TritonX-100 for 10 min at room temperature. Cells were then washed once in DPBS and once in Wash Buffer (2× SSC buffer, 10% formamide) before incubation with 20 ng antisense FISH probe in a fixation buffer overnight at 50 °C. Samples were subsequently washed twice for 5 min with Wash Buffer at 50 °C and mounted on coverslips with mounting medium containing DAPI. Imaging was performed on a Nikon SoRa Spinning Disk Confocal CSU-W1 microscope, and data analyzed using ImageJ v1.54f.

### 2.9. Poly(dT) Pulldowns of Aβ-GFP

4 × 10^6^ HeLa cells were electroporated with either 30 μg Aβ-GFP or 10 μg GFP expression vector and seeded into 10 cm dishes (one transfection per dish). Then, 24 h after transfection, cells were washed twice with ice-cold DPBS and resuspended in 300 μL lysis buffer (10 mM Tris HCl pH 7.4, 100 mM KCl, 5 mM MgCl_2_, 0.5% sodium deoxycholate, 1% Triton X-100) supplemented with Phosphatase inhibitor cocktail 2 (1:100) and RNasin (1:1000). After incubation on ice for 15 min, lysates were centrifuged at 10,000× *g* for 5 min at 4°C. Protein concentrations of the supernatants were measured and normalized. A total of 350 μL lysate was added to 50 μL of washed Oligo (dT)_25_ Magnetic beads (New England Biolabs, Ipswich, MA, USA) and incubated for 30 min at room temperature. Beads were washed three times with a lysis buffer and resuspended in 10 μL lysis buffer containing 250 units of Benzonase for elution. After 15 min at 37 °C, the supernatant was collected and analyzed by SDS-PAGE and Western blotting with anti-GFP antibody.

### 2.10. Quantitative Mass Spectrometry

*Sample preparation*. WT or TRMT61A KD cells were transfected by electroporation with either Aβ-EGFP or EGFP. 24 h after transfection, cells were washed with PBS, resuspended in 300 μL lysis buffer, and kept on ice for 20 min. After centrifugation at 10,000× *g* for 5 min at 4 °C, the supernatants were collected and normalized for protein concentration. Each sample was then incubated with 10 μL GFP-Trap Magnetic Agarose (ChromoTek, Planegg-Martinsried, Germany) for 1 h at 4 °C. The agarose was washed three times with lysis buffer and three times with MS buffer (50 mM Tris HCl pH 7.4, 150 mM NaCl). The agarose was resuspended in 50 μL 8 M urea/50 mM Tris HCl pH 8.5, reduced with 10 mM DTT for 30 min, and alkylated with 40 mM chloroacetamide for 20 min at 22 °C. Urea was diluted to a final concentration of 2 M with 25 mM Tris HCl pH 8.5, 10% acetonitrile and proteins were digested with trypsin/lys-C mix overnight at 22 °C. Acidified peptides (0.1% trifluoroacetic acid) were desalted and fractionated on combined C18/SCX stage tips (3 fractions). For peptide fractionation, self-packed stage tips were prepared using Empore C18-PS solid-phase extraction (SPE) disks and Empore PK20 cation-exchange SPE disks (both from 3M, St. Paul, MN, USA). After trypsin/Lys-C digestion, peptide samples were loaded onto C18 stage tips, washed, and then eluted directly onto the cation-exchange stage tips using 80% acetonitrile (ACN)/0.5% acetic acid. Peptides bound to the cation-exchange stage tips were subsequently eluted in three fractions sequentially applying 50 mM ammonium acetate/20% ACN/0.5% formic acid, 150 mM ammonium acetate/20% ACN/0.5% formic acid, and 5% ammonium hydroxide/80% ACN. Peptides were dried and resolved in 1% acetonitrile and 0.5% formic acid.

*LC-MS/MS.* LC-MS/MS was performed on a Q Exactive Plus mass spectrometer equipped with an ultra-high-pressure liquid chromatography unit (Easy-nLC1000) and a Nanospray Flex Ion-Source (all from Thermo Fisher Scientific). Peptides were separated on an in-house packed column (100 μm inner diameter, 30 cm length, 2.4 μm Reprosil-Pur C18 resin) using a gradient from mobile phase A (4% acetonitrile, 0.1% formic acid) to 30% mobile phase B (80% acetonitrile, 0.1% formic acid) for 60 min followed by a second step to 60% B for 30 min, with a flow rate of 300 nl/min. MS data were acquired in data-dependent mode, selecting the 10 most abundant precursor ions for HCD fragmentation with a normalized collision energy of 30. The full MS scan range was set from 300 to 2000 *m*/*z* with a resolution of 70,000. Ions with a charge ≥2 were selected for MS/MS scan with a resolution of 17,500 and an isolation window of 2 *m*/*z*. The maximum ion injection time for the survey scan and the MS/MS scans was 120 ms, and the ion target values were set to 3 × 10^6^ and 10^5^, respectively. The dynamic exclusion of selected ions was set to 30 s. Data were acquired using Xcalibur software (Thermo Fisher Scientific).

*Data analysis using MaxQuant*. MS raw files from five biological replicates of pulldown and background samples were analyzed using Max Quant (version 1.5.3.30) [15] applying default parameters. Enzyme specificity was set to trypsin and lysC, allowing up to two missed cleavages. A minimal peptide length of seven amino acids was required. Carbamidomethylcysteine was set as a fixed modification, whereas N-terminal acetylation and methionine oxidation were set as variable modifications. Spectra were searched against the UniProtKB human FASTA database (downloaded November 2015, 70,075 entries) for protein identification with a false discovery rate of 1%. Unidentified features were matched between runs within a 2 min retention time window. In cases where identified peptides were shared between multiple proteins, they were combined and reported as protein groups. Hits in three categories (false positives, only identified by site, and known contaminants) were excluded from further analysis. For label-free quantification (LFQ), the minimum ratio count was set to 1. Data analysis was conducted using Perseus. Bioinformatic data analysis was performed using Perseus (version 1.5.2.6) [16]. Proteins identified in the pulldown experiments were included if quantified in at least 4 of 5 biological replicates in at least one group (pulldown or background). Missing LFQ values were imputed on the basis of a normal distribution (width of 0.3, downshift of 1.3). Proteins enriched in the pulldown were identified using a two-sample *t*-test with a permutation-based FDR cut-off of 0.05 and s0 = 0.1. Categorical annotations were added in Perseus, and Fisher’s exact test with a *p*-value threshold of 0.001 was performed for GO term enrichment analysis.

## 3. Results

### 3.1. Deficiency of TRMT61A Enhances Amyloidogenesis

To investigate the role of m^1^A during proteostasis stress, we generated TRMT61A knockdown (KD) HeLa cells using CRISPR-Cas9. TRMT61A encodes the catalytic subunit of the N1-adenine methyltransferase complex TRMT6/61A responsible for installing m^1^A [17]. Complete deletion of TRMT61A is not feasible, as it is among the core essential genes required for human cell survival [18]. The aneuploid HeLa cell line, with up to six copies of each chromosome [19], provides a convenient experimental tool for achieving partial gene inactivation. Partial deletion of TRMT61A in HeLa cells was successful in the past and resulted in decreased cellular m^1^A levels [10]. Figure S1 shows the residual TRMT61A protein levels in our KD cells. The remaining methylation of tRNA in KD cells is sufficient to support translation in our experimental model, as protein synthesis and cell viability in knockdown cells were not significantly affected, and proliferation was only slightly slower, as we reported previously [10].

**Figure 1:**
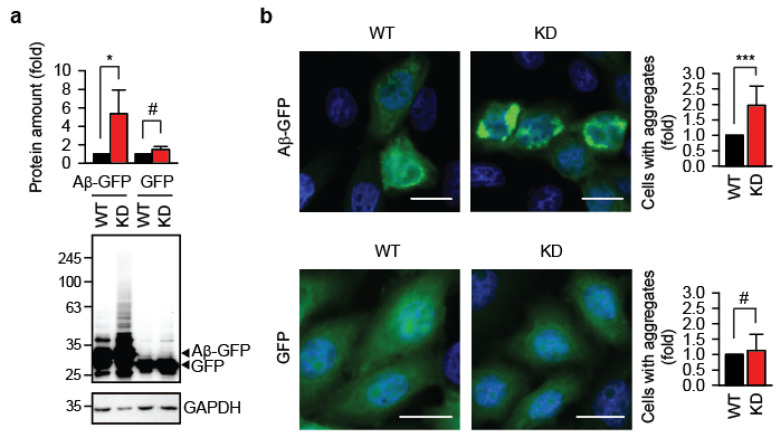
**Knockdown of the N1-adenine methyltransferase TRMT61A enhances amyloid accumulation.** (**a**) Quantification of the Aβ-GFP or GFP accumulation in HeLa WT or TRMT61A KD cells by Western blotting. The lower arrow indicates GFP. GAPDH was used as a loading control. * *p* < 0.05; #, not significant; two-tailed *t*-test; N = 3 independent experiments (mean ± SD). (**b**) Fluorescent microscopy images and aggregate quantification in Aβ-GFP- or GFP-overexpressing HeLa WT and TRMT61A KD cells. Scale bar: 20 µm. *** *p* < 0.001, chi-square test (N = 3, mean ± SD). Blue, DAPI; green, GFP.

Using the TRMT61A KD cells, we assessed whether defective N1-adenine methylation affects protein aggregation. To this end, we transiently expressed amyloid-β 1–42 fused to GFP (Aβ-GFP) in wild-type (WT) and TRMT61A KD HeLa cells. Aβ1–42′s strong amyloidogenic properties are well established for modeling amyloid fibril formation [20]. Western blot analysis 48 h after transfection revealed significantly higher accumulation of Aβ-GFP in KD cells (Figure 1a). Amyloid oligomers are known to inhibit protein degradation by the proteasome [21], which could explain the accumulation of Aβ-GFP. In turn, increased levels of Aβ-GFP would shift the folding–misfolding equilibrium toward amyloid formation, as amyloidogenesis is concentration-dependent [22]. GFP, which lacks the aggregation-prone Aβ region, does not initiate this vicious cycle. Fluorescence microscopy confirmed enhanced Aβ-GFP aggregation in TRMT61A-deficient cells (Figure 1b, upper panel). Control cells transfected with GFP alone showed minimal aggregation with no difference between WT and KD lines (Figure 1b, lower panel). Importantly, similar accumulation of GFP in wild-type and knockdown cells argues against general impairment of the protein translation machinery due to TRMT61A deficiency in these experimental settings.

**Figure 2:**
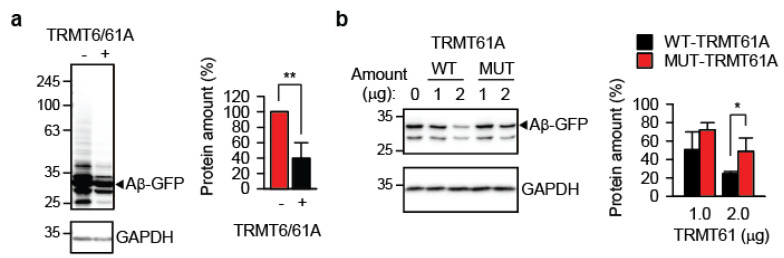
**Reintroduction of enzymatically active TRMT61A suppresses amyloid accumulation.** (**a**) Western blot analysis of overexpressed Aβ-GFP in TRMT61A knockdown cells. TRMT6 and TRMT61A were co-transfected as indicated. GAPDH was used as loading control. ** *p* < 0.01, two-tailed *t*-test; N = 3 independent experiments (mean ± SD). (**b**) An activity-impaired TRMT61A mutant (MUT-TRMT61A) fails to suppress the accumulation of Aβ-GFP as efficiently as the wild-type enzyme (WT-TRMT61A) in TRMT61A knockdown HeLa cells. Cells were analyzed by Western blot 24 h after transfection. GAPDH was used as a loading control. * *p* < 0.05, two-tailed *t*-test (N = 4, mean ± SD).

Reintroducing the catalytically active TRMT6/61A heterodimer into TRMT61A KD cells significantly reduced Aβ-GFP accumulation (Figure 2a). In contrast, the enzymatically impaired TRMT61A mutant D181A was much less effective (Figure 2b), demonstrating that TRMT61A’s catalytic activity is required to suppress amyloid accumulation. This result indicates that RNA binding alone by the TRMT6/61A is not sufficient for suppression of amyloid formation; the m^1^A modification itself is required for protection. The expression levels of wild-type and enzymatically impaired FLAG-tagged TRMT61A proteins were comparable (Figure S2).

To support this conclusion, an orthogonal approach of reducing the intracellular amount of m^1^A was used: instead of diminishing the activity of the N1-adenine methyltransferase, the m^1^A demethylase ALKBH3 was overexpressed [23]. Immunofluorescence revealed enhanced accumulation of Aβ-GFP aggregates in these cells, recapitulating the effect of TRMT61A depletion (Figure S3).

### 3.2. The m^1^A Methylation Motif Protects Transcript Function During Amyloidogenesis

**Figure 3:**
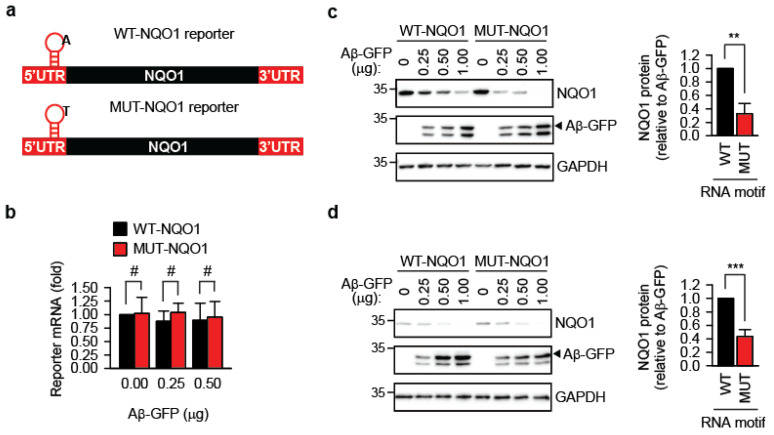
**Reporter containing the m^1^A methylation motif produces more protein during amyloidogenesis.** (**a**) Schematic representation of the m^1^A motif-containing NQO1 reporters. UTR, untranslated region. The position of the motif on the transcript is indicated. The nucleotide sequence of the motif is shown in Figure S4a. (**b**) Reporter mRNA levels do not differ significantly after 24 h of co-expression with Aβ-GFP, as determined by quantitative PCR. #, not significant; two-tailed *t*-test; N = 3 independent experiments (mean ± SD). (**c**) Accumulation of NQO1 protein from WT-NQO1 and MUT-NQO1 transcripts 24 h after co-transfection with the indicated amounts of Aβ-GFP. One representative anti-FLAG Western blot (NQO1) is shown. GAPDH was used as a loading control. For quantitative comparison, the NQO1 amount per unit of Aβ-GFP was calculated and averaged for all three Aβ-GFP concentrations. This value for WT-NQO1 reporter was set as 1. ** *p* < 0.01, two-tailed *t*-test; N = 4 independent experiments (mean ± SD). (**d**) Accumulation of NQO1 protein from WT-NQO1 and MUT-NQO1 transcripts 24 h after co-transfection with the indicated amounts of Aβ-GFP in murine melanoma B16-F10 cells. One representative anti-FLAG Western blot (NQO1) is shown. GAPDH was used as a loading control. For quantitative comparison, the NQO1 amount per unit of Aβ-GFP was calculated and averaged for all three Aβ-GFP concentrations. This value for WT-NQO1 reporter was set as 1. *** *p* < 0.001, two-tailed *t*-test; N = 4 independent experiments (mean ± SD).

Previously, we showed that m^1^A-modified transcripts are protected during acute proteostasis stress induced by heat shock [10]. Here, we tested whether this protective effect extends to amyloidogenesis. A short TRMT6/61A-sensitive m^1^A motif derived from the 5′-UTR of the PRUNE transcript [8,24] was inserted 12 nucleotides upstream of the start codon of the human NAD(P)H:quinone oxidoreductase 1 gene, generating the wild-type reporter WT-NQO1 (Figure 3a and Figure S4a). To distinguish reporter-expressed NQO1 from endogenous NQO1, an N-terminal 3xFLAG tag was included. As a control, the mutant reporter MUT-NQO1 was constructed, in which the methylation-sensitive adenine in the m^1^A motif was replaced with thymidine (Figure 3a and Figure S4a). qPCR showed that both reporters were expressed at similar RNA levels upon transfection into HeLa cells (Figure 3b).

Transient co-expression in wild-type HeLa cells of the reporters and Aβ-GFP led to reduced accumulation of the FLAG-NQO1 protein, inversely correlating with Aβ-GFP amyloid levels (Figure 3c). Notably, this reduction was significantly greater for MUT-NQO1, suggesting that m^1^A methylation in the TRMT6/61A motif protects the transcript’s function in the presence of amyloid. This difference between WT-NQO1 and MUT-NQO1 reporters in the presence of Aβ-GFP could be reproduced in another cell line, the murine melanoma B16-F10 (Figure 3d).

Given the similar steady-state RNA levels of WT-NQO1 and MUT-NQO1 in the cell (Figure 3b), the difference in protein output could result from altered interactions of the wild-type versus mutant motifs independent of methylation. To directly test whether methylation is required for the protective effect, we examined NQO1 accumulation in TRMT61A KD HeLa cells. In these methylation-deficient cells, WT-NQO1 and MUT-NQO1 produced similar protein levels, indicating that TRMT6/61A-mediated methylation is essential for the protective advantage of the WT-NQO1 reporter (Figure S4b).

A straightforward explanation for these observations is that methylation status affects transcript entanglement with amyloid aggregates. Transcripts trapped in aggregates would be sequestered from the translation machinery, reducing protein synthesis. However, attempts to experimentally confirm this by localizing WT-NQO1 and MUT-NQO1 transcripts in aggregates using RNA FISH were unsuccessful (Figure S5). Technical challenges associated with the analyzes of transcripts potentially entrapped in amyloid mandate cautious interpretation of this negative result, as discussed later.

### 3.3. TRMT61A Deficiency Increases the Association of RBPs with Amyloid

**Figure 4:**
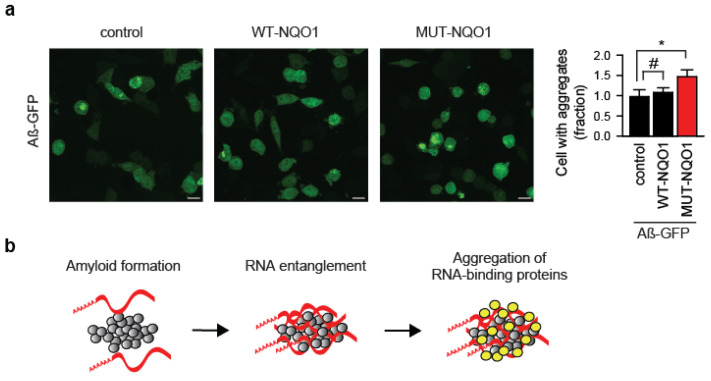
**Protein aggregation driven by reporter RNA expression.** (**a**) Immunofluorescent images and quantification of Aβ–GFP aggregation in HeLa WT cells co-transfected with either wild-type (WT-NQO1) or mutant (MUT-NQO1) reporter. Equal amounts of Aβ-GFP and NQO1 reporter plasmids (15 μg each) were electroporated. Scale bar: 20 µm. Fractions of amyloid-containing cells were quantified and compared (right panel). * *p* < 0.05, #, not significant; two-tailed *t*-test; N = 3 independent experiments (mean ± SD). (**b**) Model of RNA-driven aggregation of RNA-binding proteins with amyloid. Red strips, mRNA; gray circles, amyloid; yellow circles, RNA-binding proteins.

Unexpectedly, overexpression of the MUT-NQO1 reporter alone was sufficient to enhance Aβ-GFP aggregation in wild-type HeLa cells (Figure 4a). This suggests that the inability to safeguard even a single RNA species from amyloid entanglement can significantly exacerbate protein aggregation. A plausible explanation involves a molecular “chain reaction”, where amyloid-trapped RNA recruits bystander RNA-binding proteins (RBPs), which then aberrantly sequester additional mRNAs into the aggregates (Figure 4b).

**Figure 5:**
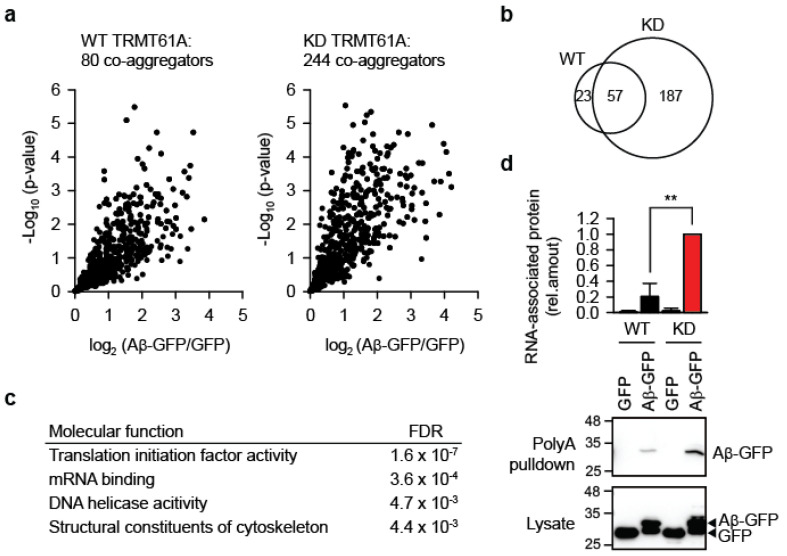
**Aggregation of bystander proteins and mRNAs is increased under the defective N1-adenine methylation.** (**a**) Mass spectrometry analysis of proteins co-aggregating with Aβ-GFP in WT or TRMT61A-deficient HeLa cells 24 h after transfection. Volcano plot showing quantified proteins plotted according to their enrichment on Aβ-GFP over GFP background; statistical significance of the respective ratios is plotted on the y-axis. The size of each sets is indicated. N = 5 independent experiments. (**b**) Overlap between Aβ-GFP co-aggregators in WT and TRMT61A KD cells. (**c**) GO Molecular function categories enriched in the Aβ-GFP co-aggregator set in TRMT61A KD cells, but not in co-aggregators in WT cells. (**d**) Increased amyloid association with mRNAs in TRMT61A KD cells 24 h after Aβ-GFP transfection, as determined by poly(dT) pulldowns. Transfection of GFP was used as a control. ** *p* < 0.01, two-tailed *t*-test; N = 3 independent experiments (mean ± SD).

Beyond biomembrane damage, another important component of the amyloid toxicity is the co-aggregation of bystander proteins. Highly toxic amyloid variants have been shown to entrap and co-aggregate more cellular proteins than their less toxic forms [25,26]. We therefore asked whether impaired RNA homeostasis due to insufficient N1-adenine methylation increases bystander protein aggregation. To test this, we overexpressed Aβ-GFP in WT and TRMT61A KD HeLa cells and analyzed aggregates by quantitative mass spectrometry 24 h post-transfection. In KD cells, amyloid aggregates contained three times as many bystander proteins compared to WT (Figure 5a, Tables S1 and S2). Specifically, we identified 244 proteins significantly enriched in KD aggregates versus 80 proteins in aggregates from wild-type cells. Although there was considerable overlap between co-aggregomes (Figure 5b), unique categories emerged in KD cells’ aggregates, including four functional categories absent from WT (Figure 5c). The top-two category was “mRNA binding”, supporting the idea that amyloid-associated RNAs drive protein co-aggregation through recruitment of their cognate RBPs (Figure 4b).

Finally, to directly test whether m^1^A insufficiency increases mRNA association with aggregates, we used poly(dT)-beads to isolate polyadenylated RNAs from WT and KD cells after Aβ-GFP expression. The experiment revealed significantly greater mRNA association with amyloid in KD cells (Figure 5d). Note that Aβ-GFP accumulation differs between Figure 1a (two days post-transfection) and Figure 5d (one day post-transfection)—for the mass spectrometry and RNA association analyses an earlier time point was selected to bias for interactions with potentially causal relevance during amyloidogenesis.

## 4. Discussion

N1-methyladenine (m^1^A) has been suggested to safeguard tagged mRNAs during acute proteostasis stress, such as heat or arsenite exposure [10]. The current study extends these previous observations by demonstrating that modulation of the m^1^A-installing machinery exacerbates transcriptome damage under chronic proteotoxic stress. Specifically, we found that reducing m^1^A levels—either by TRMT61A knockdown or ALKBH3 overexpression in HeLa cells expressing Aβ1–42—led to significantly enhanced protein aggregation, impaired translation of a reporter mRNA, and increased accumulation of RNAs and RNA-binding proteins (RBPs) in amyloid inclusions. These findings suggest that m^1^A can partially protect against aberrant RNA–protein interactions in cells experiencing sustained amyloid stress. This protective role mirrors m^1^A’s function during acute proteotoxicity: under heat shock, TRMT6/61A and m^1^A accumulate in stress granules, and introducing an m^1^A consensus motif into a reporter RNA improves recovery of translation from that reporter upon return to normal temperature [10]. Similarly, in the current study, during chronic amyloidogenic stress, m^1^A-marked transcripts produced more protein compared to their A-to-T mutated counterparts (Figure 3), suggesting improved protection from irreversible sequestration in aggregates.

What could underlie the surprisingly strong impact of a small methyl group on the intracellular behavior of vastly larger mRNA molecules? Two possibly overlapping scenarios are conceivable: (1) adenine methylation-induced changes in global RNA structure; and/or (2) local alterations in hydrogen bonding potential of methyladenine. N1-methylation of adenine introduces a positive charge and blocks both Watson–Crick and Hoogsteen base-pairing, which can strongly affect inter- and intramolecular interactions and the formation of structural elements such as RNA hairpins [27,28]. By perturbing RNA structure, m^1^A may reduce an mRNA’s propensity to form stable contacts with RBPs or other RNAs. This aligns with growing evidence for the role of nucleic acids in protein aggregation. For example, RNA has been shown to accelerate α-synuclein fibrillization, with fibrillar α-synuclein sequestering large amounts of RNA via electrostatic interactions [29]. Similarly, another study demonstrated that RNA concentration modulates hnRNP1A aggregation: at low RNA levels, condensation and fibril formation were promoted, whereas at high levels, condensates dissolved and amyloid formation slowed [30].

Alternatively, even if the gross RNA structure remains unchanged, local disruption of hydrogen bonding upon adenine methylation may alter interactions with dedicated m^1^A “reader” proteins. Such reader proteins are thought to mediate specific functions associated with RNA modifications [31]. Recently, significant progress in understanding structural and functional details in this regard has been achieved, especially for N6-methyladenine. The identification of m^1^A readers has been hampered due to the chemical instability of this modification, which manifests as the Dimroth rearrangement, i.e., the conversion of m^1^A to m^6^A. In spite of the technical difficulties, several m^1^A readers have been identified (YTHDF1-3 and YTHDC1) [32] and verified using rigorous controls (YTHDF1 and YTHDF2) [33] and it is quite probable that more will be discovered in the future. However, none of these were detected among aggregate-associated proteins (Table S2). This conforms to our model in which the absence of the m^1^A tag would preclude association of RNAs with their cognate m^1^A readers, leaving transcripts more susceptible to aberrant entanglement with misfolding and aggregating proteins during proteostasis stress. In turn, RNAs sequestered in aggregates could recruit bystander RBPs, amplifying proteome damage (Figure 4b). Our mass spectrometry data revealed significant enrichment of RBPs in TRMT6/61A deficient cells, supporting the model (Figure 5c). Understanding of the relative importance of m^1^A readers in the observed enhancement of aggregation will require biochemical identification of endogenous transcripts in aggregates and possibility to verify them as TRMT6/61A substrates.

Technical challenges associated with analyzing cellular aggregates must be considered when interpreting negative data. We invested substantial effort to localize WT-NQO1 and MUT-NQO1 reporter transcripts in amyloid using FISH but were unsuccessful (Figure S5). At least two explanations are possible: first, interactions between reporter transcripts and amyloid might suffice to impair translation without being strong or stable enough for detection under our experimental conditions; second, entanglement with amyloid may shield sequences required for probe hybridization. A potential solution would be biochemical isolation and identification of endogenous transcripts present in aggregates, enabling the selection of transcripts more abundantly incorporated into amyloid and thus more likely to be detected by microscopy. Moreover, exploring different sequences may be necessary to identify transcripts compatible with probe hybridization within amyloid.

Recently, it was shown that adenine in CAG-repeat RNA can be methylated to N1-methyladenine by TRMT61A and demethylated by ALKBH3, with m^1^A levels correlating with CAG repeat length [34]. TDP-43 bound directly and strongly with m^1^A-labeled CAG-RNA, which led to the cytosolic mislocalization and aggregation of TDP-43. TDP-43 is an RBP that aggregates in the cytosol of the degenerating neurons in amyotrophic lateral sclerosis (ALS) patients [35]. The enhanced protein aggregation due to m^1^A labeling is an opposite effect what was observed in our study. However, the molecular setups differ fundamentally. Expanded CAG RNAs are known to undergo phase transitions forming repetitive homomeric structures in vitro and RNA foci in human cells [36]. It is conceivable that aggregation-prone m^1^A interactors, such as TDP-43, when recruited to such structures are forced to oligomerize and aggregate. In contrast, our study addressed a broad spectrum of normal mRNAs, each carrying only a single m^1^A label and encountering preformed amyloid. In other words, m^1^A can play different roles: it promotes the aggregation of some proteins when present on CAG-repeat RNAs many times but protects against aggregation when installed only once on conventional transcripts.

In summary, we demonstrate that N1-adenine methylation machinery protects the cellular proteome during amyloid formation. This novel role of m^1^A—buffering RNA-mediated interactions during amyloidogenesis—complements the recently reported function of m^1^A in acute stress granules [10]. Our findings supports the notion that dynamic RNA methylation contributes to cellular proteostasis by modulating RNA–protein interactions involved in the formation of physiological and pathological aggregates.

## Data Availability

The data that support the findings of this study are available from the corresponding author upon reasonable request. The mass spectrometry proteomics data have been deposited to the ProteomeXchange Consortium via the PRIDE [37] partner repository with the dataset identifier PXD016658.

## Supplementary Materials

The following supporting information can be downloaded at https://www.mdpi.com/article/10.3390/cells14171370/s1; Figure S1: HeLa knockdown cells; Figure S2: Overexpression of TRMT61A; Figure S3: Overexpression of the N1-methyladenine demethylase ALKBH3; Figure S4: Reporter containing the m^1^A-motif; Figure S5: Localization of NQO1 reporter RNAs in transiently transfected HeLa cells; Table S1: MaxQLF quantitative data; Table S2: Aß-GFP interactors.

## Abbreviations

The following abbreviations are used in this manuscript:

Aβ_1–42_	Amyloid-β peptide 1–42
ALKBH3	AlkB homolog 3
ALS	amyotrophic lateral sclerosis
DAPI	4′,6-diamidino-2-phenylindole
FISH	fluorescence in situ hybridization
FTO	Fat mass and obesity-associated protein
GAPDH	Glyceraldehyde 3-phosphate dehydrogenase
GFP	Green fluorescent protein
hnRNPA1	Heterogeneous Nuclear Ribonucleoprotein A1
KD	knockdown
LFQ	label-free quantification
m^1^A	N^1^-methyladenine
MS	mass spectrometry
NQO1	NAD(P)H Quinone dehydrogenase 1
PFA	paraformaldehyde
RBP	RNA-binding protein
SAM	S-adenosyl methionine
SG	stress granule
TDP-43	Transactive response DNA binding protein 43 kDa
TRMT6	tRNA methyltransferase 6 non-catalytic subunit
TRMT61A	tRNA methyltransferase 61A
UTR	untranslated region
WT	wild-type

## References

[1] Balch W.E., Morimoto R.I., Dillin A., Kelly J.W. (2008). Adapting Proteostasis for Disease Intervention. Science.

[2] Cappannini A., Ray A., Purta E., Mukherjee S., Boccaletto P., Moafinejad S.N., Lechner A., Barchet C., Klaholz B.P., Stefaniak F. (2024). MODOMICS: A Database of RNA Modifications and Related Information. 2023 Update. Nucleic Acids Res..

[3] McCown P.J., Ruszkowska A., Kunkler C.N., Breger K., Hulewicz J.P., Wang M.C., Springer N.A., Brown J.A. (2020). Naturally Occurring Modified Ribonucleosides. Wiley Interdiscip. Rev. RNA.

[4] RajBhandary U.L., Stuart A., Faulkner R.D., Chang S.H., Khorana H.G. (1966). Nucleotide Sequence Studies on Yeast Phenylalanine sRNA. Cold Spring Harb. Symp. Quant. Biol..

[5] Anderson J., Phan L., Cuesta R., Carlson B.A., Pak M., Asano K., Björk G.R., Tamame M., Hinnebusch A.G. (1998). The Essential Gcd10p–Gcd14p Nuclear Complex Is Required for 1-Methyladenosine Modification and Maturation of Initiator Methionyl-tRNA. Genes Dev..

[6] Nguyen N.Y.T., Liu X., Dutta A., Su Z. (2025). The Secret Life of N1-Methyladenosine: A Review on Its Regulatory Functions. J. Mol. Biol..

[7] Fedeles B.I., Singh V., Delaney J.C., Li D., Essigmann J.M. (2015). The AlkB Family of Fe(II)/α-Ketoglutarate-Dependent Dioxygenases: Repairing Nucleic Acid Alkylation Damage and Beyond. J. Biol. Chem..

[8] Safra M., Sas-Chen A., Nir R., Winkler R., Nachshon A., Bar-Yaacov D., Erlacher M., Rossmanith W., Stern-Ginossar N., Schwartz S. (2017). The m1A Landscape on Cytosolic and Mitochondrial mRNA at Single-Base Resolution. Nature.

[9] Li X., Xiong X., Wang K., Wang L., Shu X., Ma S., Yi C. (2016). Transcriptome-Wide Mapping Reveals Reversible and Dynamic N1-Methyladenosine Methylome. Nat. Chem. Biol..

[10] Alriquet M., Calloni G., Martínez-Limón A., Delli Ponti R., Hanspach G., Hengesbach M., Tartaglia G.G., Vabulas R.M. (2020). The Protective Role of m1A during Stress-Induced Granulation. J. Mol. Cell Biol..

[11] Ramaswami M., Taylor P.J., Parker R. (2013). Altered Ribostasis: RNA-Protein Granules in Degenerative Disorders. Cell.

[12] Zhang X., Lin Y., Eschmann N.A., Zhou H., Rauch J.N., Hernandez I., Guzman E., Kosik K.S., Han S. (2017). RNA Stores Tau Reversibly in Complex Coacervates. PLoS Biol..

[13] van den Hoff M.J., Moorman A.F., Lamers W.H. (1992). Electroporation in “intracellular” Buffer Increases Cell Survival. Nucleic Acids Res..

[14] Livak K.J., Schmittgen T.D. (2001). Analysis of Relative Gene Expression Data Using Real-Time Quantitative PCR and the 2^−ΔΔCT^ Method. Methods.

[15] Cox J., Mann M. (2008). MaxQuant Enables High Peptide Identification Rates, Individualized p.p.b.-Range Mass Accuracies and Proteome-Wide Protein Quantification. Nat. Biotechnol..

[16] Tyanova S., Temu T., Sinitcyn P., Carlson A., Hein M.Y., Geiger T., Mann M., Cox J. (2016). The Perseus Computational Platform for Comprehensive Analysis of (Prote)Omics Data. Nat. Methods.

[17] Ozanick S., Krecic A., Andersland J., Anderson J.T. (2005). The Bipartite Structure of the tRNA m1A58 Methyltransferase from S. Cerevisiae Is Conserved in Humans. RNA.

[18] Blomen V.A., Májek P., Jae L.T., Bigenzahn J.W., Nieuwenhuis J., Staring J., Sacco R., van Diemen F.R., Olk N., Stukalov A. (2015). Gene Essentiality and Synthetic Lethality in Haploid Human Cells. Science.

[19] Landry J.J.M., Pyl P.T., Rausch T., Zichner T., Tekkedil M.M., Stütz A.M., Jauch A., Aiyar R.S., Pau G., Delhomme N. (2013). The Genomic and Transcriptomic Landscape of a HeLa Cell Line. G3.

[20] Lee D.Y., Lee K.-S., Lee H.J., Kim D.H., Noh Y.H., Yu K., Jung H.-Y., Lee S.H., Lee J.Y., Youn Y.C. (2010). Activation of PERK Signaling Attenuates Aβ-Mediated ER Stress. PLoS ONE.

[21] Tseng B.P., Green K.N., Chan J.L., Blurton-Jones M., LaFerla F.M. (2008). Abeta Inhibits the Proteasome and Enhances Amyloid and Tau Accumulation. Neurobiol. Aging.

[22] Ke P.C., Zhou R., Serpell L.C., Riek R., Knowles T.P.J., Lashuel H.A., Gazit E., Hamley I.W., Davis T.P., Fändrich M. (2020). Half a Century of Amyloids: Past, Present and Future. Chem. Soc. Rev..

[23] Aas P.A., Otterlei M., Falnes P.O., Vågbø C.B., Skorpen F., Akbari M., Sundheim O., Bjørås M., Slupphaug G., Seeberg E. (2003). Human and Bacterial Oxidative Demethylases Repair Alkylation Damage in Both RNA and DNA. Nature.

[24] Li X., Xiong X., Zhang M., Wang K., Chen Y., Zhou J., Mao Y., Lv J., Yi D., Chen X.-W. (2017). Base-Resolution Mapping Reveals Distinct m1A Methylome in Nuclear- and Mitochondrial-Encoded Transcripts. Mol. Cell.

[25] Olzscha H., Schermann S.M., Woerner A.C., Pinkert S., Hecht M.H., Tartaglia G.G., Vendruscolo M., Hayer-Hartl M., Hartl F.U., Vabulas R.M. (2011). Amyloid-like Aggregates Sequester Numerous Metastable Proteins with Essential Cellular Functions. Cell.

[26] Kim Y.E., Hosp F., Frottin F., Ge H., Mann M., Hayer-Hartl M., Hartl F.U. (2016). Soluble Oligomers of PolyQ-Expanded Huntingtin Target a Multiplicity of Key Cellular Factors. Mol. Cell.

[27] Zhou H., Kimsey I.J., Nikolova E.N., Sathyamoorthy B., Grazioli G., McSally J., Bai T., Wunderlich C.H., Kreutz C., Andricioaei I. (2016). m1A and m1G Potently Disrupt A-RNA Structure Due to the Intrinsic Instability of Hoogsteen Base Pairs. Nat. Struct. Mol. Biol..

[28] Micura R., Pils W., Höbartner C., Grubmayr K., Ebert M.O., Jaun B. (2001). Methylation of the Nucleobases in RNA Oligonucleotides Mediates Duplex-Hairpin Conversion. Nucleic Acids Res..

[29] Rupert J., Monti M., Zacco E., Tartaglia G.G. (2023). RNA Sequestration Driven by Amyloid Formation: The Alpha Synuclein Case. Nucleic Acids Res..

[30] Morelli C., Faltova L., Capasso Palmiero U., Makasewicz K., Papp M., Jacquat R.P.B., Pinotsi D., Arosio P. (2024). RNA Modulates hnRNPA1A Amyloid Formation Mediated by Biomolecular Condensates. Nat. Chem..

[31] Flamand M.N., Tegowski M., Meyer K.D. (2023). The Proteins of mRNA Modification: Writers, Readers, and Erasers. Annu. Rev. Biochem..

[32] Dai X., Wang T., Gonzalez G., Wang Y. (2018). Identification of YTH Domain-Containing Proteins as the Readers for N1-Methyladenosine in RNA. Anal. Chem..

[33] Seo K.W., Kleiner R.E. (2020). YTHDF2 Recognition of N1-Methyladenosine (m1A)-Modified RNA Is Associated with Transcript Destabilization. ACS Chem. Biol..

[34] Sun Y., Dai H., Dai X., Yin J., Cui Y., Liu X., Gonzalez G., Yuan J., Tang F., Wang N. (2023). m1A in CAG Repeat RNA Binds to TDP-43 and Induces Neurodegeneration. Nature.

[35] Portz B., Lee B.L., Shorter J. (2021). FUS and TDP-43 Phases in Health and Disease. Trends Biochem. Sci..

[36] Jain A., Vale R.D. (2017). RNA Phase Transitions in Repeat Expansion Disorders. Nature.

[37] Perez-Riverol Y., Bandla C., Kundu D.J., Kamatchinathan S., Bai J., Hewapathirana S., John N.S., Prakash A., Walzer M., Wang S. (2025). The PRIDE Database at 20 Years: 2025 Update. Nucleic Acids Res..

