# Supplementary material for: Deficiency of N1-Adenine Methyltransferase Aggravates RNA and Protein Aggregation
Source: Cells. 2025 Sep 2;14(17):1370. doi: 10.3390/cells14171370 (PMC12428715; doi:10.3390/cells14171370)
Supplement: Supplementary file 1 [file cells-14-01370-s001.zip › Figure S5.pdf]

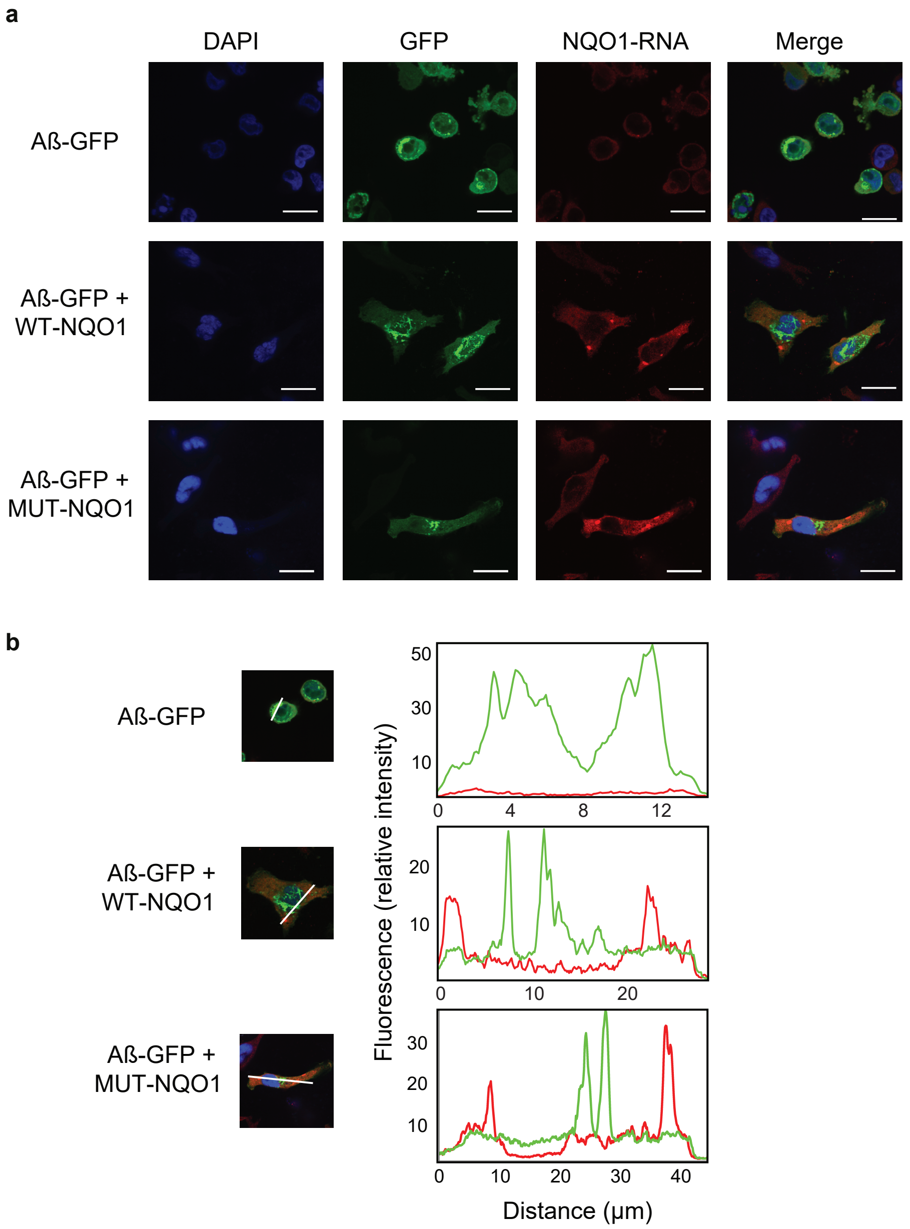

Figure S5. Localization of NQO1 reporter RNAs in transiently transfected HeLa cells. (a) Representative RNA FISH images of HeLa WT cells co-transfected with NQO1 reporters. Blue, DAPI; green, GFP; red, NQO1 RNA. Scale bar: 20 µm. (b) Line plots of fluorescence intensity for the green (Aß-GFP) and red (NQO1 RNA) channels in individual cells, corresponding to the panels shown on the left.
